# Evaluation of Influenza A H1N1 infection and antiviral utilization in a tertiary care hospital

**DOI:** 10.1186/s12879-018-3492-z

**Published:** 2018-11-16

**Authors:** Talita Rantin Belucci, Alexandre R. Marra, Michael B. Edmond, João Renato Rebello Pinho, Paula Kiyomi Onaga Yokota, Ana Carolina Cintra Nunes Mafra, Oscar Fernando Pavão dos Santos

**Affiliations:** 1 Hospital Israelita Albert Einstein, São Paulo, Brazil; 2 Division of Medical Practice, Hospital Israelita Albert Einstein, Avenida Albert Einstein, 627 – bloco A1, 1° andar, Morumbi, São Paulo, 05651-901 Brazil; 3 Office of Clinical Quality, Safety and Performance Improvement, University of Iowa Hospitals and Clinics, Iowa City, IA USA; 4 Division of Infectious Diseases, Department of Internal Medicine, University of Iowa Carver College of Medicine, Iowa City, IA USA; 5 Clinical Laboratory, Hospital Israelita Albert Einstein, São Paulo, Brazil; 6 Statistics Department, Instituto Israelita de Ensino e Pesquisa Albert Einstein, Hospital Israelita Albert Einstein, São Paulo, Brazil; 7 Núcleo de Indicadores e Sistemas de Informações, Hospital Israelita Albert Einstein, São Paulo, Brazil; 8 LIM 03/07, Faculdade de Medicina da USP, São Paulo, Brazil

**Keywords:** Influenza a H1N1, Oseltamivir, Hospitalized patients

## Abstract

**Background:**

Influenza A H1N1 infections carry a significant mortality risk. This study describes inpatients with suspected and confirmed Influenza A H1N1 infection who were prescribed oseltamivir, the risk factors associated with infection, the association between infection and mortality, and the factors associated with in-hospital mortality in infected patients.

**Methods:**

This study was a matched case-control study of hospitalized patients who underwent real-time polymerase chain reaction testing for Influenza A H1N1 and were treated with oseltamivir from 2009 to 2015 in a tertiary care hospital. Cases (patients with positive Influenza A H1N1 testing) were matched 1:1 to controls (patients with negative test results).

**Results:**

A total of 1405 inpatients who underwent PCR testing and received treatment with oseltamivir were identified in our study and 157 patients confirmed Influenza A H1N1. Almost one third of patients with Influenza A H1N1 were diagnosed in the pandemic period. There was no difference in mortality between cases and controls. Immunocompromised status, requirement of vasoactive drugs, mechanical ventilation, acute hemodialysis, albumin administration, surgical procedures and thoracic procedures and length of stay were associated with increased risk of death in Influenza A H1N1 infected patients.

**Conclusions:**

We found no increased risk of mortality for patients with proven Influenza A H1N1 when compared to similar patients without confirmed Influenza.

**Electronic supplementary material:**

The online version of this article (10.1186/s12879-018-3492-z) contains supplementary material, which is available to authorized users.

## Background

According to the World Health Organization (WHO), during the Influenza A H1N1 pandemic 59 million people were infected, resulting in 265,000 hospitalizations and 12,000 deaths in the United States. This virus has high transmissibility, a short incubation period, and high rates of morbidity and mortality [1].

The goal of this study is to describe inpatients treated with oseltamivir and suspected and confirmed Influenza A H1N1 infection, the associated factors with infection, the association between infection and mortality, and the factors associated with in-hospital mortality in patients with confirmed Influenza A H1N1.

## Methods

This study was conducted in a tertiary care, private hospital in São Paulo, Brazil with 629 beds and approximately 194,000 patient-days yearly and approved by the Institutional Review Board and Ethics Committee of Hospital Israelita Albert Einstein and informed consent was not required.

A retrospective study was conducted from January 2009 to December 2015.

This study describes inpatients treated with oseltamivir who had suspected or confirmed Influenza A H1N1 infection and were tested for Influenza A H1N1 by real-time polymerase chain reaction (RT-PCR). The primary reason for hospitalization was not necessarily Influenza. This study also describes the factors associated with infection, the association between infection and mortality and the factors associated with in-hospital mortality in patients with confirmed Influenza A H1N1.

A matched (1:1) case-control study was performed to analyze the factors associated with infection and in-hospital mortality, comparing patients who would have similar illness severity during hospitalization and thus isolate the impact of the infection on the outcome of in-hospital mortality. Cases were defined as patients with Influenza A H1N1 confirmed by RT-PCR, and controls had a negative result for Influenza A (H1N1 and H3N2) and Influenza B and were treated with oseltamivir for up to four days. All patients in the matched case-controls study were tested both for Influenza A H1N1 and Influenza A H3N2, but only 31.6% (444/1.405) were tested for Influenza B. Patients excluded were those under 18 years of age and those in whom the length of stay exceeded 365 days. The criteria of length of stay was based on the long-term hospitalized patients.

The data abstracted from the electronic medical record included demographics and clinical data, Influenza RT-PCR assay results, oseltamivir treatment (duration, frequency and dose), outcome status (death was defined as in-hospital mortality), and underlying conditions (lung disease, cardiovascular disease, neurological and neuro-developmental conditions, blood disorders, diabetes mellitus, kidney disease, liver disease, immunosuppression (e.g., HIV, cancer or chronic treatment with corticosteroids), and pregnancy or post-partum state [up to two weeks after childbirth]). We also collected possible indicators of complications during hospitalization (which may or may not be associated with H1N1 infection): data on intensive care unit (ICU) admission, transfusions, use of mechanical ventilation, acute hemodialysis, use of vasopressor drugs, albumin and antibiotic administration, surgical procedures, or thoracic procedures (e.g., pulmonary biopsy or segmentectomy, tracheostomy).

Patients were not followed up after hospital discharge. Antiviral therapy was prescribed according to the institutional protocol [2], with oseltamivir initiated empirically based on clinical presentation or after a positive PCR test. The empiric therapy for Influenza is based on the symptoms, such as fever, cough, sore throat, runny and/or stuffy nose, muscle or body aches, headaches, and fatigue, as well as for patients at high risk for developing Influenza- related complications. This includes age > 60 years, patients of any age with certain chronic medical conditions (such as cardiovascular disease, lung disease, diabetes mellitus, kidney disease, liver disease, neurological and neuro-developmental conditions, and immunocompromised states), and pregnant women or post-partum state [1, 2].

### Statistical analysis

Descriptive analysis was performed using the median and inter-quartile range (IQR) for continuous variables and absolute frequencies and percentages for categorical variables. Simple associations were analyzed using logistic models and odds ratio were determined. The level of significance was set at 0.05.

For matching cases and controls we used the Matching package [3], which weighs all variables involved in order to have a balanced final pairing. Cases and controls were matched on factors impacting mortality: age, ICU admission, surgical procedure, use of vasoactive drugs, use of mechanical ventilation, albumin administration, and blood or platelet transfusion. After matching, the logistic model predicting death was adjusted by means of generalized estimation equations, with the Geepack package [4]. We used R software version 3.4.1.

## Results

**Fig. 1:**
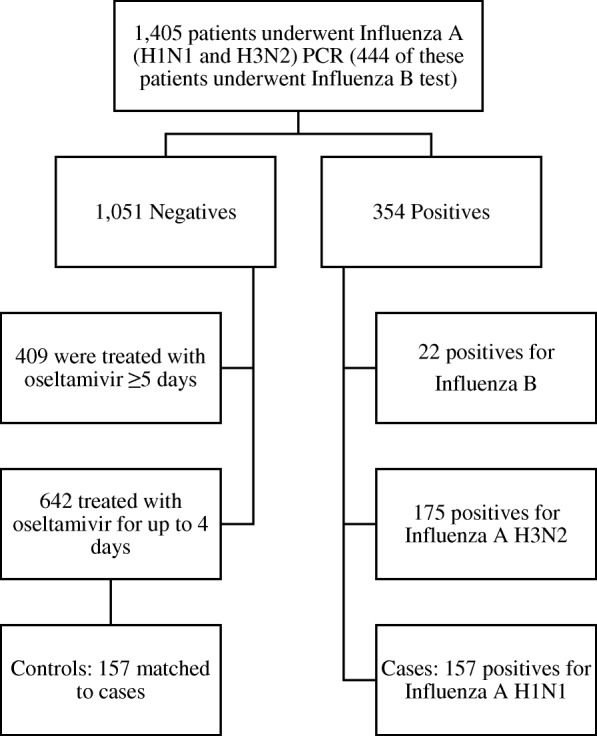
Patients included in the matched (1:1) case-control study

Of 1,405 inpatients who underwent PCR testing and received treatment with oseltamivir, 1051 (74.8%) were PCR negative. Twenty-two patients were positive for Influenza B, 175 positive for Influenza A H3N2, and 157 positive for Influenza A H1N1. Of the uninfected patients, 642 received oseltamivir treatment up to four days and 157 of those were matched as controls. For the 157 controls, 32 (20.4%) underwent Influenza B testing and were also negative for Influenza B (Fig. 1).

When considering the entire period of the study, 19.2% of the requests for PCR tests combined with oseltamivir prescriptions occurred during the period of the pandemic, with the majority (60.7%) occurring between 2013 and 2015. These occurred in older patients (55.1% > 60 years), 49.9% males, 1.9% pregnant or post-partum state, 8.1% immunocompromised, 23.7% with diabetes mellitus, 24.3% with lung disease, 1.2% with liver disease, 6.0% with kidney disease, 46.8% with cardiovascular disease and 6.8% with neurological and neuro-developmental conditions (Additional file 1: Table S1). The primary diagnosis was a disease of the respiratory system in 66.6% of hospitalizations (Additional file 1: Table S1).

Of the 157 Influenza A H1N1 cases, 49.7% (78/157) were diagnosed in the pandemic period. In 85.4% (134/157) of the patients with Influenza A H1N1 infection, the daily dosage of oseltamivir was 150 mg and 92.4% (145/157) were treated for 5–10 days (Table 1).

**Table 1:** Characteristics of inpatients with suspected Influenza (*N* = 1208)

	Influenza status	
Variable	Infected	Uninfected
	(*n* = 157)	(*n* = 1051)
Period – *n* (%)		
2009 (Pandemic period)	78 (49.7)	185 (17.6)
2010 to 2012	12 (7.7)	200 (19.0)
2013 to 2015	67 (42.7)	666 (63.4)
Oseltamivir daily dosage – *n* (%)		
75 mg	–	2 (0.2)
150 mg	134 (85.4)	925 (88.0)
300 mg	23 (14.6)	124 (11.8)
Oseltamivir treatment duration (days) – *n* (%)		
< 5 days	–	642 (61.1)
5 to 10 days	145 (92.4)	405 (38.5)
> 10 days	12 (7.6)	4 (0.4)

Category variables presented by absolute and relative frequencies. Numerical variables presented by median and inter-quartile range

From 2013, the number of hospitalizations increased, especially in the uninfected group (666/1051) (Table 1). Infected patients were 2.86 fold more likely to be immunocompromised (*P* = 0.033) and one-third less likely to receive antibacterial therapy (*P* = 0.005) when compared to similar patients without confirmed Influenza (Table 2).

**Table 2:** Patients profile and associated factors with H1N1 infection based on matched case-control study (*n* = 314)

	Influenza status			
Variable	Infected	Uninfected	OR (95% CI)	*P* value
	(*n* = 157)	(*n* = 157)		
Characteristics of patients				
Male	85 (54.1)	83 (52.9)	1.05 (0.68–1.64)	0.821
Age (> 60 years old)	37 (23.6)	58 (36.9)	–	–
Cardiovascular disease	54 (34.4)	49 (31.2)	1.16 (0.72–1.85)	0.548
Lung disease	31 (19.7)	42 (26.8)	0.67 (0.39–1.14)	0.143
Diabetes mellitus	28 (17.8)	28 (17.8)	1.00 (0.56–1.79)	1.000
Pregnant or post-partum state	11 (7.0)	4 (2.5)	2.88 (0.96–10.58)	0.075
Immunocompromised	16 (10.2)	6 (3.8)	2.86 (1.14–8.15)	0.033
Kidney disease	11 (7.0)	6 (3.8)	1.90 (0.70–5.63)	0.219
Liver disease	1 (0.6)	3 (1.9)	0.329 (0.016–2.602)	0.338
Neurological and neuro-developmental conditions	8 (5.1)	11 (7.0)	0.71 (0.27–1.81)	0.479
Hospitalization characteristics				
Antibacterial therapy (intravenous or oral)	135 (86.0)	150 (95.5)	0.29 (0.11–0.66)	0.005
ICU stay	62 (39.5)	74 (47.1)	0.73 (0.47–1.14)	0.172
Mechanical ventilation	17 (10.8)	17 (10.8)	–	–
Acute hemodialysis	8 (5.1)	0 (0.0)	–	–
Vasoactive drugs	20 (12.7)	20 (12.7)	–	–
Red blood cell or platelet transfusion	4 (2.5)	4 (2.5)	–	–
Surgical procedure	15 (9.6)	15 (9.6)	–	–
Thoracic procedure	6 (3.8)	0 (0.0)	–	–
Albumin administration	23 (14.6)	24 (15.3)	–	–
Length of stay (days)	5.00 [3.00; 8.00]	5.00 [3.00, 10.00]	1.00 (0.98–1.01)	0.680
In–hospital death	8 (5.1)	14 (8.9)	0.55 (0.21–1.32)	0.190

Category variables presented by absolute and relative frequencies. Numerical variables presented by median and inter-quartile range. Odds ratio (OR), confidence interval (95% CI) and *p* value obtained by simple logistic regression

Table 2 also shows for those patients with Influenza A H1N1 infection, 86% (135/157) were also treated with antibacterials. The median of length of stay was 5 days, and 39.5% (62/157) of the patients were admitted to the ICU. Eight patients (5.1%) required acute hemodialysis and six patients (3.8%) underwent a thoracic procedure (lung biopsy, lung segmentectomy, thoracostomy with closed drainage, drainage of chest wall hematoma, or tracheostomy).

Of patients with Influenza A H1N1, 5.1% died. Independent factors associated with mortality were requirement for vasoactive drugs (OR = 17.13, IC 95%: 5.28–55.59, *P* < 0.001), and length of stay (OR = 1.03, IC 95%: 1.01–1.06, *P* = 0.010), and when controlling for these two factors, infection with Influenza A H1N1 was not an independent predictor of mortality (OR = 0.45, CI 95%: 0.15–1.35, *P* = 0.154) (Table 3).

**Table 3:** Independent predictors of death in inpatients with suspected Influenza A H1N1 (*n* = 314)

Variable	OR (95% CI)	*P* value
Influenza A H1N1 infection	0.45 (0.15; 1.35)	0.154
Administration of vasoactive drugs	17.13 (5.28; 55.59)	< 0.001
Length of stay (days)	1.03 (1.01; 1.06)	0.010

*OR* odds ratio. CI: 95% confidence interval

When considering only the 157 patients with Influenza A H1N1 infection, the associated factors with death were: immunocompromised state (*P* = 0.019), requirement for vasoactive drugs (*P* < 0.001), mechanical ventilation (*P* < 0.001), acute hemodialysis (*P* = 0.024), surgical procedure (*P* < 0.001), thoracic surgery (*P* < 0.001), albumin administration (*P* < 0.001), and length of stay (*P* < 0.001) (Table 4).

**Table 4:** Univariate predictors of death in patients with confirmed Influenza A H1N1 infection (*n* = 157)

Variable	Discharge (*n* = 149)	Death (*n* = 8)	OR (95% CI)	*P* value
Male gender	79 (53.0)	6 (75.0)	2.66 (0.59; 18.55)	0.240
Age (> 60 years old)	35 (23.5)	2 (25.0)	1.09 (0.15; 4.96)	0.922
Immunocompromised status	13 (8.7)	3 (37.5)	6.28 (1.18; 28.72)	0.019
Diabetes mellitus	25 (16.8)	3 (37.5)	2.98 (0.58; 12.94)	0.153
Kidney disease	9 (6.0)	2 (25.0)	5.19 (0.69; 26.60)	0.063
Cardiovascular disease	50 (33.6)	4 (50.0)	1.98 (0.45; 8.69)	0.348
Hospitalization characteristics				
Vasoactive drugs	14 (9.4)	6 (75.0)	28.93 (6.04; 210.94)	< 0.001
Mechanical ventilation	12 (8.1)	5 (62.5)	19.03 (4.18; 102.52)	< 0.001
Acute hemodialysis	6 (4.0)	2 (25.0)	7.94 (1.02; 44.30)	0.024
Surgical procedure	10 (6.7)	5 (62.5)	23.17 (5.00; 127.25)	< 0.001
Thoracic surgery	3 (2.0)	3 (37.5)	29.20 (4.49; 199.40)	< 0.001
Albumin administration	17 (11.4)	6 (75.0)	23.29 (4.93; 168.04)	< 0.001
Length of stay (days)	5 [3, 7]	38 [11.5, 53.75]	1.07 (1.03; 1.11)	< 0.001

Category variables presented by absolute and relative frequencies. Numerical variables presented by median and inter-quartile range. *OR* odds ratio. *95% CI* 95% confidence interval

## Discussion

We observed a high proportion of negative PCRs among patients treated with oseltamivir because the treatment was administered empirically based on symptoms. The recommendation is to initiate the treatment with oseltamivir within 48 h of Influenza symptom onset [5], especially in critically ill patients, in order to reduce symptom duration, complications such as pneumonia, and possibly death [6]. However, empiric therapy leads to uninfected patients receiving treatment and testing modalities other than PCR often have low sensitivity and specificity [7, 8].

This study considered the RT-PCR test as a gold standard, but other tests, such Influenza A and B by immunofluorescence, Influenza A serology, Influenza B serology, Rapid Influenza A and B diagnostic test, screening for respiratory virus (Influenza A and B) by immunofluorescence, viral culture (Influenza A and B), may have been conducted during the study period.

In our study, surgical procedures and thoracic procedures were associated with an increased risk of death in Influenza A H1N1 patients, but we did not find others studies that analyzed surgical procedures in inpatients with Influenza A H1N1 infection.

More than 80% of the patients with Influenza A H1N1 in our study were treated with antibacterials, which in some cases was due to pneumonia complicating Influenza. In our study it was not possible to identify if the patients had pneumonia, but in a large cohort study [9] 31% of the patients with Influenza A H1N1 infection were diagnosed with bacterial pneumonia.

We observed that the number of hospitalizations increased substantially from 2013 onward. This rise can be explained by the fact that in that period a viral panel performed by PCR method was introduced in the hospital’s testing routine, and beyond Influenza A H1N1, the panel could also identify Influenza A H3N2 and Influenza B. That is why the information on Influenza B is not available for all patients in the study. From 2013 to 2015, the most prevalent Influenza virus in the Southeast region of Brazil, (where the hospital is located) was Influenza A H3N2 [10–12].

In the matched case-controls study results, there was no difference in mortality between patients with and without Influenza infection. However, it should be noted that all patients were treated with oseltamivir. This finding reinforces the need for treatment within 48 h of symptom onset, even in those patients who are not at high risk of developing Influenza–related complications [1, 5].

In our study, only 8 (5.1%) patients with Influenza A H1N1 infection died. Immunocompromised states were associated with mortality in patients with Influenza A H1N1 infection in our study and in a Spanish study in which 25% (68/274) of immunocompromised inpatients with Influenza A H1N1 infection died [13]. A study performed in immunosuppressed patients with Influenza A H1N1 admitted to the ICU concluded that this population has a poor outcome and the use of corticosteroids is strongly discouraged [14].

Patients that require vasoactive drugs and mechanical ventilation were also at increased risk of death from Influenza A H1N1 infection. In the same Spanish study, 78.7% (214/274) Influenza A H1N1 infected patients requiring vasoactive drugs and 92.2% (249/274) requiring mechanical ventilation also died [13]. The median length of stay for inpatients who died in our study was much higher than that observed in this same study (13 days) [13].

Another Spanish study found that chronic conditions were an independent risk factor for mortality [15]; we noted a similar trend in our study that was not statistically significant.

Our study has some limitations, primarily that it is single center and retrospective. The retrospective nature of the study made it impossible to identify the exact cause of pneumonia. We also cannot attribute the cause of death to Influenza infection. Our study evaluated only patients tested by PCR and were treated with oseltamivir. The patients were not followed after discharge, so it was only possible to identify in-hospital deaths. It was also not possible to check the patient’s vaccination status.

## Conclusion

In conclusion, the profiles of the infected and uninfected patients were very similar and there was no difference in mortality. The only risk factor associated with death in infected patients was an immunocompromised state.

## Additional file

Additional file 1: **Table S1.** Profile of inpatients suspected for Influenza A H1N1 infection, with oseltamivir prescription and underwent real-time polymerase chain reaction (RT-PCR). (DOCX 17 kb)

## Abbreviations

CI: Confidence intervals
ICU: Intensive care unit
IQR: Inter-quartile range
OR: Odds ratios
RT-PCR: Real-time polymerase chain reaction
WHO: World Health Organization
