# Supplementary material for: Evaluation of Influenza A H1N1 infection and antiviral utilization in a tertiary care hospital
Source: BMC Infect Dis. 2018 Nov 16;18:579. doi: 10.1186/s12879-018-3492-z (PMC6240327; doi:10.1186/s12879-018-3492-z)
Supplement: Supplementary file 1 — Table S1. Profile of inpatients suspected for Influenza A H1N1 infection, with oseltamivir prescription and underwent real-time polymerase chain reaction (RT-PCR). (DOCX 17 kb) [file 12879_2018_3492_MOESM1_ESM.docx]

**Additional file 1: Table S1.** Profile of inpatients suspected for Influenza A H1N1 infection, with oseltamivir prescription and underwent real-time polymerase chain reaction (RT-PCR).

| Characteristics of patients | Negative and oseltamivir  ≤ 4 days | Negative and oseltamivir  > 4 days | Influenza B | Influenza A H1N1 | Influenza A H3N2 | Overall |
| --- | --- | --- | --- | --- | --- | --- |
| *n* | *642* | *409* | *22* | *157* | *175* | *1405* |
| Period |  |  |  |  |  |  |
| 2009 (Pandemic period) | 79 (12.3) | 106 (25.9) | 0 (0.0) | 78 (49.7) | 7 (4.0) | 270 (19.2) |
| 2010 to 2012 | 133 (20.7) | 67 (16.4) | 0 (0.0) | 12 (7.6) | 70 (40.0) | 282 (20.1) |
| 2013 to 2015 | 430 (67.0) | 236 (57.7) | 22 (100.0) | 67 (42.7) | 98 (56.0) | 853 (60.7) |
| Male | 337 (52.5) | 187 (45.7) | 11 (50.0) | 85 (54.1) | 81 (46.3) | 701 (49.9) |
| Age (> 60 years old) | 391 (60.9) | 232 (56.7) | 14 (63.6) | 37 (23.6) | 100 (57.1) | 774 (55.1) |
| Pregnant or post-partum state | 7 (1.1) | 3 (0.7) | 0 (0.0) | 11 (7.0) | 5 (2.9) | 26 (1.9) |
| Immunocompromised | 51 (7.9) | 22 (5.4) | 2 (9.1) | 16 (10.2) | 23 (13.1) | 114 (8.1) |
| Diabetes mellitus | 161 (25.1) | 103 (25.2) | 3 (13.6) | 28 (17.8) | 38 (21.7) | 333 (23.7) |
| Lung disease | 159 (24.8) | 109 (26.7) | 5 (22.7) | 31 (19.7) | 38 (21.7) | 342 (24.3) |
| Liver disease | 12 (1.9) | 3 (0.7) | 0 (0.0) | 1 (0.6) | 1 (0.6) | 17 (1.2) |
| Kidney disease | 36 (5.6) | 30 (7.3) | 0 (0.0) | 11 (7.0) | 7 (4.0) | 84 (6.0) |
| Cardiovascular disease | 307 (47.8) | 210 (51.3) | 11 (50.0) | 54 (34.4) | 76 (43.4) | 658 (46.8) |
| Neurological and neuro-developmental conditions | 54 (8.4) | 25 (6.1) | 0 (0.0) | 8 (5.1) | 8 (4.6) | 95 (6.8) |
| Primary diagnosis of diseases of the respiratory system (ICD-10 J00-J99) | 398 (62.0) | 280 (68.5) | 14 (63.6) | 115 (73.2) | 129 (73.7) | 936 (66.6) |
